# Improved Protocol for DNA Extraction from Subsoils Using Phosphate Lysis Buffer

**DOI:** 10.3390/microorganisms8040532

**Published:** 2020-04-07

**Authors:** Victor Guerra, Lukas Beule, Ena Lehtsaar, Hui-Ling Liao, Petr Karlovsky

**Affiliations:** 1 North Florida Research and Education Center, Soil and Water Sciences Department, University of Florida, Quincy, FL 32351, USA; v.guerra@ufl.edu (V.G.); sunny.liao@ufl.edu (H.-L.L.); 2 Molecular Phytopathology and Mycotoxin Research, Faculty of Agricultural Sciences, University of Goettingen, 37075 Goettingen, Germany; ena.lehtsaar@stud.uni-goettingen.de (E.L.); pkarlov@gwdg.de (P.K.)

**Keywords:** soil DNA extraction, subsoil, clayey soils, soil bacteria, soil fungi, inter-laboratory comparison

## Abstract

As our understanding of soil biology deepens, there is a growing demand for investigations addressing microbial processes in the earth beneath the topsoil layer, called subsoil. High clay content in subsoils often hinders the recovery of sufficient quantities of DNA as clay particles bind nucleic acids. Here, an efficient and reproducible DNA extraction method for 200 mg dried soil based on sodium dodecyl sulfate (SDS) lysis in the presence of phosphate buffer has been developed. The extraction protocol was optimized by quantifying bacterial 16S and fungal 18S rRNA genes amplified from extracts obtained by different combinations of lysis methods and phosphate buffer washes. The combination of one minute of bead beating, followed by ten min incubation at 65°C in the presence of 1 M phosphate buffer with 0.5% SDS, was found to produce the best results. The optimized protocol was compared with a commonly used cetyltrimethylammonium bromide (CTAB) method, using Phaeozem soil collected from 60 cm depth at a conventional agricultural field and validated on five subsoils. The reproducibility and robustness of the protocol was corroborated by an interlaboratory comparison. The DNA extraction protocol offers a reproducible and cost-effective tool for DNA-based studies of subsoil biology.

## 1. Introduction

In agricultural systems, the distinction between top- and subsoil is made based on the present or historical tillage depth, which is commonly around 20 to 30 cm [1,2]. Although subsoil accounts for the vast majority of agricultural soil [2] and its management becomes increasingly important in light of resource scarcity, [3] our knowledge on soil processes and microbial communities in subsoils is scarce compared to topsoils. Fortunately, however, the number of scientific studies investigating both top- and subsoils is steadily increasing, as the methodologies for such studies become more refined and reliably reproducible. These investigations have broadened the understanding of the role of soil microbes in carbon and nutrient cycling, as well as the fate of soil pollutants [4]. There is a growing body of literature recognizing the significance of microbial processes influencing the stability of soil organic carbon in subsoils [5,6,7,8]. Physio-chemical properties have been reported to vary with soil depth [9,10,11], and a greater spatial heterogeneity has been found in subsoils [12,13] as compared to topsoils where soil is homogenized by tillage [14]. The abundance of soil microorganisms is commonly decreasing with increasing soil depth [9,15,16]. Alongside with absolute changes with increasing soil depth, compositional differences between top- and subsoil microbial communities have been reported using molecular tools [17,18]. Given the low abundance of microorganisms inhabiting subsoil environments, the extraction of sufficient amounts of nucleic acids for molecular methods like real-time PCR (qPCR) and next generation sequencing is challenging, particularly in clay-rich soil [16,19,20,21,22].

It has long been established that clays have a strong capacity to adsorb nucleic acids and nucleotides [23]. The main mechanisms contributing to the difficulty in obtaining sufficient DNA yields from clay soils are related to electrostatic and physical properties of clay minerals and nucleic acids. Cations associated with clay minerals, namely Ca^2+^ Mg^2+^ Fe^3+^ and Al^3+^, facilitate the adsorption or precipitation of DNA from solution through interactions with negatively charged phosphate groups. Expansive clays like montmorillonite intercalate a mass of DNA in excess of their own weight below pH 5 [24,25]. The effect of pH on adsorption of DNA onto clay minerals is significant, with the greatest capacity for adsorption occurring at a pH below 5.5 [24,25,26]. The fragment size of DNA is another influential factor affecting its adsorption to clay minerals [27,28]. Orgam and co-workers [27] assessed the effect of polymer length on soil adsorption in various soil types using calf thymus DNA and found that, in a silty clay soil dominated by smectite minerals, the shortest length polymer of 2.69 kbp exhibited the highest observed Freundlich adsorption coefficient (K) of the study, in excess of 300. The authors posed that such a high K value for the short fragments may be explained by their nonexclusion from the pores of the expanding clay and also by kinetics of adsorption, by which the short fragments are able to outcompete longer fragments for binding sites, due to their higher diffusion rate in intraparticle pores or water film surrounding particles [27]. The above described properties of clay and DNA present challenges that any soil DNA extraction method must overcome through effective pre-lysis and lysis treatment.

Soil DNA extractions are commonly performed using either commercial soil DNA extraction kits or in-house methods. Commercial kits offer the advantage that they are easy to use; however, compared to in-house methods, commercial kits are expensive and most of their reagents are supplied in limited volumes with an unspecified composition. Furthermore, yield differences between commercial kits have been observed [29]. The chemical diversity of soils is tremendous and may vary over several orders of magnitude [30], which may require that a DNA extraction protocol be tailored for a particular soil [31]. Therefore, small volumes of unspecified reagents of commercial kits limit the flexibility needed to account for chemical differences among soil and/or soil depths. Assessments of soil DNA extraction protocols, which included commercial extraction kits, have demonstrated that such kits do not yield sufficient quantity or quality of DNA when utilized for soils with high clay content [22,32,33]. He and colleagues [34] found that washing of soil having high clay and iron oxide content with 0.1 M sodium phosphate buffer (PB) (pH 7.5) may be implemented to obtain high quantity and quality DNA with commercial kits. The use of phosphate-based buffer washes is effective for exchanging clay bound DNA with phosphate ions (e.g., [22]), but it also elutes humic substances and other contaminants, necessitating careful implementation [35,36].

In this study, we assessed the effectiveness of different lysis procedures in combination with different phosphate buffers for recovering nucleic acid from a range of subsoils having high clay content. Subsequently, we selected one promising lysis and phosphate buffer combination, which was optimized to reliably and reproducibly extract subsoil DNA suitable for use in DNA-based studies of microbial communities.

## 2. Materials and Methods

### 2.1. Optimization of DNA Extraction from Subsoil

#### 2.1.1. Subsoil Collection for Optimization

Subsoil for DNA extraction optimization was collected on July 20, 2019 from a Phaeozem soil at a conventional agricultural field near Dornburg, Thuringia, Germany (Table S1). A soil core (Ø 10 cm) was obtained using a steel cylinder, with a hardened steel cutting head driven into the soil by an electric caulking hammer (Makita HM1400, Makita, Fischamend, Austria). Subsoil was collected from 60 cm depth and the outer few millimetres (approximately 5 mm) of the soil core were removed to avoid a transfer of the topsoil into the subsoil material (carryover) during the sampling procedure. The subsoil was homogenized in a sterile polyethylene bag and approximately 50 g of fresh soil was transferred into a sterile 50-mL Falcon tube (SARSTEDT, Nümbrecht, Germany) and frozen at −20 °C in the field. Upon arrival at the laboratory, the subsoil sample was freeze-dried for 72 h and subsequently homogenized using a swing mill (Retsch MM400, Retsch, Haan, Germany) at 25 Hz for 1 min. Homogenized soil was stored air-tight in the dark at room temperature.

#### 2.1.2. Subsoil DNA Extraction Using CTAB Buffer

Extractions were performed using 50 and 200 mg freeze-dried soil, as previously described for the upper 5-cm topsoil by [37]. Finely ground soil was weighted into 2-mL tubes and three tungsten carbide beads (Ø 3 mm) were added. The samples were pulverized using a swing mill (Retsch MM400, Retsch, Haan, Germany) at 25 Hz for 1 min and subsequently suspended in 1 mL cetyltrimethylammonium bromide (CTAB) buffer (10 mM Tris, 20 mM ethylenediaminetetraacetic acid (EDTA), 0.02 M CTAB, 0.8 M NaCl, 0.03 M N-lauroylsarcosine, 0.13 M sorbitol, 1% (*w/v*) polyvinylpolypyrrolidone, adjusted to pH 8.0 with NaOH) with 1 µL proteinase K (20 mg/mL) and 2 µL 2-mercaptoethanol. The mixture was incubated at 42 °C and subsequently at 65 °C for 10 min each, with multiple inversions every 60 s for 5 s. After incubation, 800 µL phenol (redistilled, in TE buffer equilibrated, pH 7.5 to 8.0) were added, the mixture was thoroughly shaken, and centrifuged at 7380× *g* for 10 min. Following centrifugation, 800 µL of the supernatant was transferred into a new 2-mL tube and 1 volume (800 µL) chloroform-isoamyl alcohol (24:1 (v/v)) was added. The mixture was shaken, incubated 10 min on ice, and centrifuged at 7380× *g* for 10 min. After centrifugation, 700 µL of the supernatant was transferred to a new 1.5-mL tube, to which 1 volume (700 µL) chloroform-isoamyl alcohol (24:1 (*v/v*)) was added. The mixture was incubated for 10 min on ice and centrifuged at 7380× *g* for 10 min. Following this, 600 µL of the supernatant was transferred into a new 1.5-mL tube, containing 200 µL 30% (*w/v*) polyethylene glycol (PEG 6000) and 100 µL 5 M NaCl. The mixture was shaken, incubated at room temperature for 20 min, and centrifuged at 16,000× *g* for 20 min to pellet the DNA. The supernatant was discarded and the remaining DNA pellets were washed twice with 500 µL 80% (*v/v*) EtOH with a centrifugation step (5 min at 16,000× *g*) each time prior to discarding the supernatant. DNA pellets were dried at 30 °C for 15 min using a vacuum centrifuge and subsequently re-suspended in 50 μL of 1× TE buffer (10 mM Tris, 1 mM EDTA, adjusted to pH 8.0 with HCl). The dissolution of the pelleted DNA was facilitated by incubating the samples at 42 °C for 2 h. Finally, 3 µL of the DNA extracts were mixed with 2 μL loading buffer (100 mM EDTA, 50% (*v/v*) glycerol, 0.025% (*v/v*) bromophenol blue) and checked on 0.8% agarose gels (in 1 × TAE buffer (40 mM Tris, 20 mM sodium acetate, 1 mM Na_2_EDTA, adjusted to pH 7.6)) by using agarose gel electrophoresis (4.6 V/cm for 60 min). Agarose gels were stained with ethidium bromide solution (1 mg/L (w/v)) for 10 min and de-stained in double distilled water (ddH_2_O) for 10 min. Subsequently, the DNA was visualized by fluorescence in UV light. DNA extracts were stored at −20 °C until analysis.

#### 2.1.3. Subsoil DNA Extraction Using Phosphate Buffer

**Figure 1:**
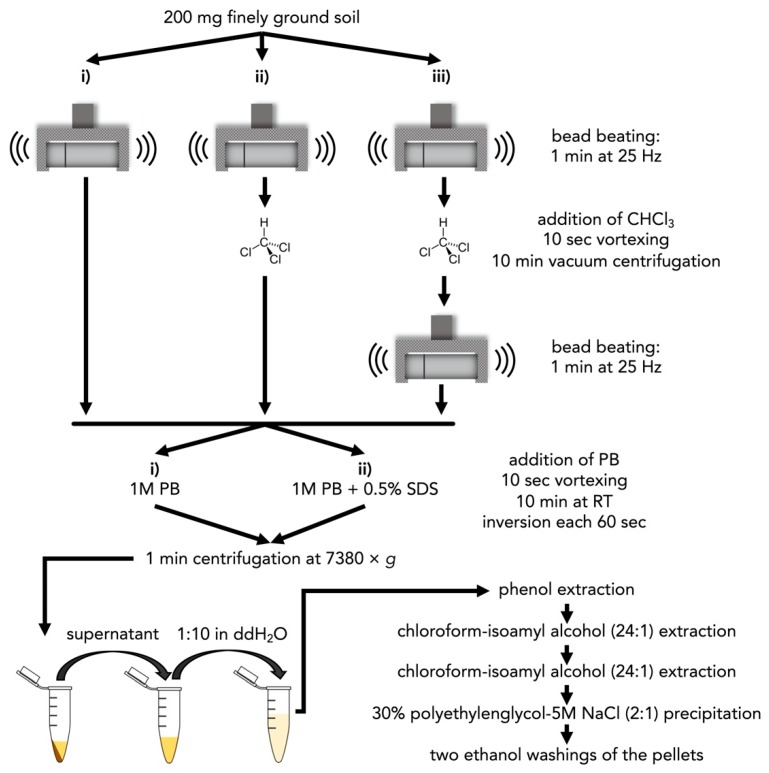
Workflow illustration of preliminary test of different combinations of lysis method and buffer solution. ddH_2_O = double-distilled water; PB = phosphate buffer; RT = room temperature; SDS = sodium dodecyl sulfate.

DNA was extracted from 200 mg of finely ground freeze-dried soil, weighted into 2-mL tubes. Microbial cells in soil were mechanically lysed by adding three tungsten carbide beads (Ø 3 mm) and bead beating the samples using a swing mill (Retsch MM400, Retsch, Haan, Germany) at 25 Hz for 1 min. Additionally, we tested whether a saturation step with chloroform, which is expected to increase the permeability of membranes of microbial cells that were not mechanically lysed, can increase DNA yield. For this, 250 μL chloroform was added to the 2-mL tubes and the suspension was vortexed for 10 s. Following this, the chloroform was completely evaporated at 30 °C for 10 min using a vacuum centrifuge. Dried soil pellets were either used directly for DNA extraction or again pulverized using a swing mill as described above prior to extraction, resulting in three cell lysis methods prior to DNA extraction: i) bead beating, ii) bead beating + chloroform, and iii) bead beating + chloroform + bead beating (Figure 1).

**Figure 2:**
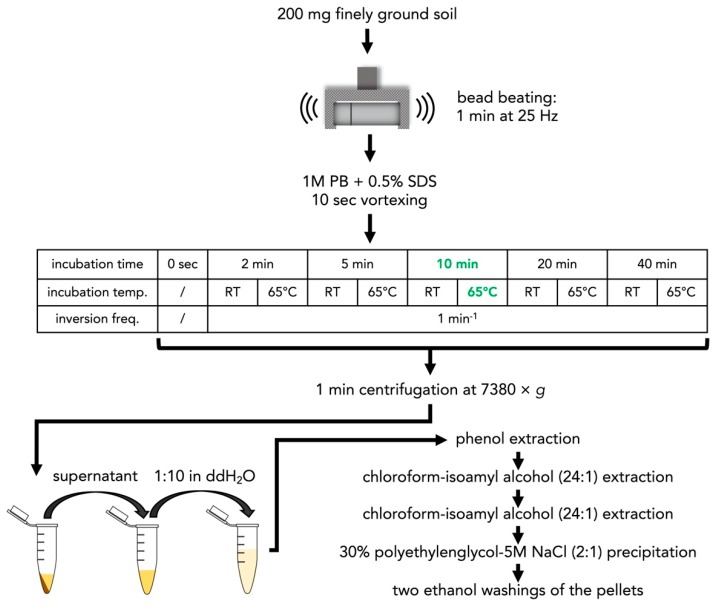
Workflow illustration of the incubation temperature and time gradient of the soil/PB with 0.5% SDS suspension. ddH_2_O = double-distilled water; PB = phosphate buffer; RT = room temperature; SDS = sodium dodecyl sulfate.

For each of these three cell lysis methods, two different phosphate buffers (PBs) were used to compete with DNA for the adsorption sites of the soil matrix: i) 1 M PB (1 M Na_2_HPO_4_ and 1 M NaH_2_PO_4_, blended to achieve pH 7.2) and ii) 1 M PB with 0.5% (*w/v*) sodium dodecyl sulfate (SDS) (Figure 2). The addition of SDS in the PB was chosen, because this anionic detergent lyses cells and denatures proteins [38], but in contrast to CTAB does not interact with DNA [39].

We added 250 μL of the respective PB (1 M PB with/without 0.5% SDS) to the samples and vortexed the suspension at 3000 rpm for 10 s using a HS120209 vortexing unit (Heathrow Scientific, Vernon Hills, USA). Samples were incubated at room temperate for 10 min, with shaking every minute for 5 s, to facilitate the desorption of DNA. Following incubation, samples were centrifuged at 7380× *g* for 1 min and 90 μL of the supernatant was transferred to a new 2-mL tube. The supernatant was diluted 1:10 by adding 810 μL ddH_2_O, as suggested by Hurt et al. [22], and extracted by adding 900 μL phenol. The mixture was shaken, centrifuged at 7380× *g* for 10 min, and 800 µL of the supernatant was transferred into a new 2-mL tube. The supernatant was extracted twice with chloroform-isoamyl alcohol, DNA was precipitated using PEG-NaCl and pelleted by centrifugation. DNA pellets were washed with ethanol twice, dried, and re-suspended in 50 μL of TE buffer, as described above for the CTAB method. Extracted DNA was visualized on agarose gels as described above (2.1.2. Subsoil DNA extraction using CTAB buffer).

#### 2.1.4. Optimization of the Incubation Temperature and Time in the Phosphate Buffer

Following the optimization of the cell lysis method and the choice of PB, we optimized the incubation temperature and time of the samples in the PB. For this, we chose cell lysis method i) (bead beating) in combination with PB with 0.5% SDS, which was as effective as the cell lysis method iii) (bead beating + chloroform + bead beating), but consumed less time and chemicals. The incubation times were 0 s, 2 min, 5 min, 10 min, 20 min, and 40 min at both RT and 65 °C, while the samples were shaken every minute for 5 s (Figure 2). Following incubation in the PB with 0.5% SDS, the samples were extracted as described above for the PB method. Extracted DNA was visualized on agarose gels, as described above for the CTAB method.

### 2.2. DNA Extraction from Different Types of Subsoil

Subsoil samples of different depths were collected from five sites in Germany from August to September 2019 (Table S1). We hereafter refer to these soil samples as subsoils 1 to 5. The subsoil samples were collected in 50-mL Falcon tubes (SARSTEDT, Nümbrecht, Germany), frozen at −20 °C in the field and freeze-dried for 72 h upon arrival in the laboratory. Following freeze-drying, the samples were finely ground and extracted using PB with 0.5% SDS, with 10 min incubation at 65 °C as described above (2.1.4. Optimization of the incubation temperature and time in the phosphate buffer). For subsoil 4, no supernatant was obtained after centrifuging the soil/PB suspension. Therefore, we increased the volume of PB added, from 250 to 500 μL. Furthermore, DNA precipitation of subsoil 4 was performed by using PEG-NaCl as for the other samples, as well as using 500 μL isopropanol instead of PEG-NaCl. For biochemical characterization of the soils, soil pH, soil organic C, and total N were determined from these samples, as described previously by Beule et al. [40].

### 2.3. Quantification of Soil Bacteria and Fungi

Soil bacteria and fungi were quantified in all soil DNA extracts, as described by Beule et al. [41] Briefly, fragments of bacterial 16S rRNA gene and fungal 18S rRNA gene were amplified from 1:20 dilutions of the soil DNA extracts in ddH_2_O in 4 μL reaction volumes in 384-well plates, using primer pair Eub338/Eub518 [42,43] for bacteria and FR1/FF390 [44] for fungi. All DNA extracts were amplified in triplicate and their mean was used for further analysis.

### 2.4. DNA Amplification Inhibition Test

The effect of PCR-inhibiting substances (e.g., phenolic compounds and humic acids co-extracted from the soil matrix) on the enzymatic amplification of DNA was quantified using a qPCR inhibition test. For this, we tested inhibition of the soil DNA extracts on the amplification of *Verticillium longisporum* VL43 by spiking the qPCR reactions with dilutions of the extracts. All qPCR reactions were performed in a CFX384 Thermocycler (Bio-Rad, Rüdigheim, Germany) in 384-well plates, with a total reaction volume of 4 μL. The mastermix was comprised of ddH_2_O; buffer (20 mM Tris-HCl, 10 mM (NH_4_)_2_SO_4_, 10 mM KCl, 2 mM MgSO_4_, 0.1% Triton^®^ X-100, pH 8.8 at 25 °C); additional 1 mM MgCl_2_ to achieve 3 mM Mg^2+^; 200 μM of each deoxyribonucleoside triphosphate (Bioline, Luckenwalde, Germany); 0.3 μM of each primer (OLG 70 (5′-CAGCG AAACG CGATA TGTAG-3′) and OLG 71 (5′-GGCTT GTAGG GGGTT TAGA-3′) [45]); 0.1X SYBR Green I solution (Invitrogen, Karlsruhe, Germany); 1 mg/mL bovine serum albumin; 0.025 u *Taq* DNA Polymerase (New England Biolabs, Beverly, Massachusetts, USA)). Positive controls contained 2 μL mastermix, 1 μL containing 10 pg/μL genomic DNA of *Verticillium longisporum* VL43 (provided by A. von Tiedemann, University of Goettingen) dissolved in 0.5 × TE buffer, and 1 μL ddH_2_O. Positive controls spiked with soil DNA extract contained 2 μL mastermix, 1 μL of a solution containing 10 pg/μL genomic DNA of *V*. *longisporum* VL43, and 1 μL of a 1:20 dilution of the soil DNA extract in ddH_2_O. Positive and positive controls spiked with soil DNA extracts were amplified in triplicate and their mean was used for further analysis. Negative controls were amplified in duplicates and contained 2 μL mastermix and 2 μL ddH_2_O. The thermocycling conditions consisted of an initial denaturation at 95 °C for 120 s, followed by 40 cycles of denaturation (94 °C, 10 s), annealing (60 °C, 15 s), and extension (68 °C, 15 s) and final extension (68 °C, 5 min). Melting curves were generated by heating the samples to 95 °C for 60 s and cooling to 55 °C for 60 s, followed by a temperature increase from 55 to 95 °C by 0.5 °C per step, with continuous fluorescence measurement. The effect of inhibitors on DNA amplification was determined by comparing the quantification cycles (C_q_) between pure *V. longisporum* DNA and *V. longisporum* DNA, spiked with soil DNA extracts.

### 2.5. Interlaboratory Comparison

The reproducibility of our optimized extraction method was tested by an interlaboratory comparison. Five different subsoil samples were extracted at the department of Molecular Phytopathology and Mycotoxin Research, University of Goettingen, Germany. The same soil samples, excluding subsoil 3 which had limited sample volume, were extracted at the North Florida Research and Education Center, University of Florida, United States of America. The extractions were carried out by a different researcher at each laboratory, following a step-by-step guide (Supplementary File S1).

### 2.6. Statistical Analysis

Data were tested for normality of distribution (Shapiro–Wilk test; ‘shapiro.test’-function in the R-package ‘stats’ version 3.4.3) and equality of variance (Levene’s test; ‘leveneTest’-function in the R-package ‘car’ version 3.0-0). If data were normally distributed and equality of variances was satisfied, student’s t-test (‘t.test’-function in the R-package ‘stats’ version 3.4.3) or a one-way analysis of variance (ANOVA) (‘anova’-function in the R-package ‘stats’ version 3.4.3) with Tukey’s honestly significant difference (HSD) post-hoc test and Holm-corrected *p*-values (‘TukeyHSD’-function in the R-package ‘stats’ version 3.4.3) was conducted. When data were not normally distributed or the variance of groups was not equal, a Mann–Whitney U test (‘wilcox.test’-function in the R-package ‘stats’ version 3.4.3) or Kruskal–Wallis test with multiple comparison extension (‘kruskalmc’-function in the R-package ‘pgirmess’ version 1.6.9) was performed. All statistical analyses were performed in R version 3.4.3 [46].

## 3. Results

### 3.1. Choice of Extraction Buffer

**Figure 3:**
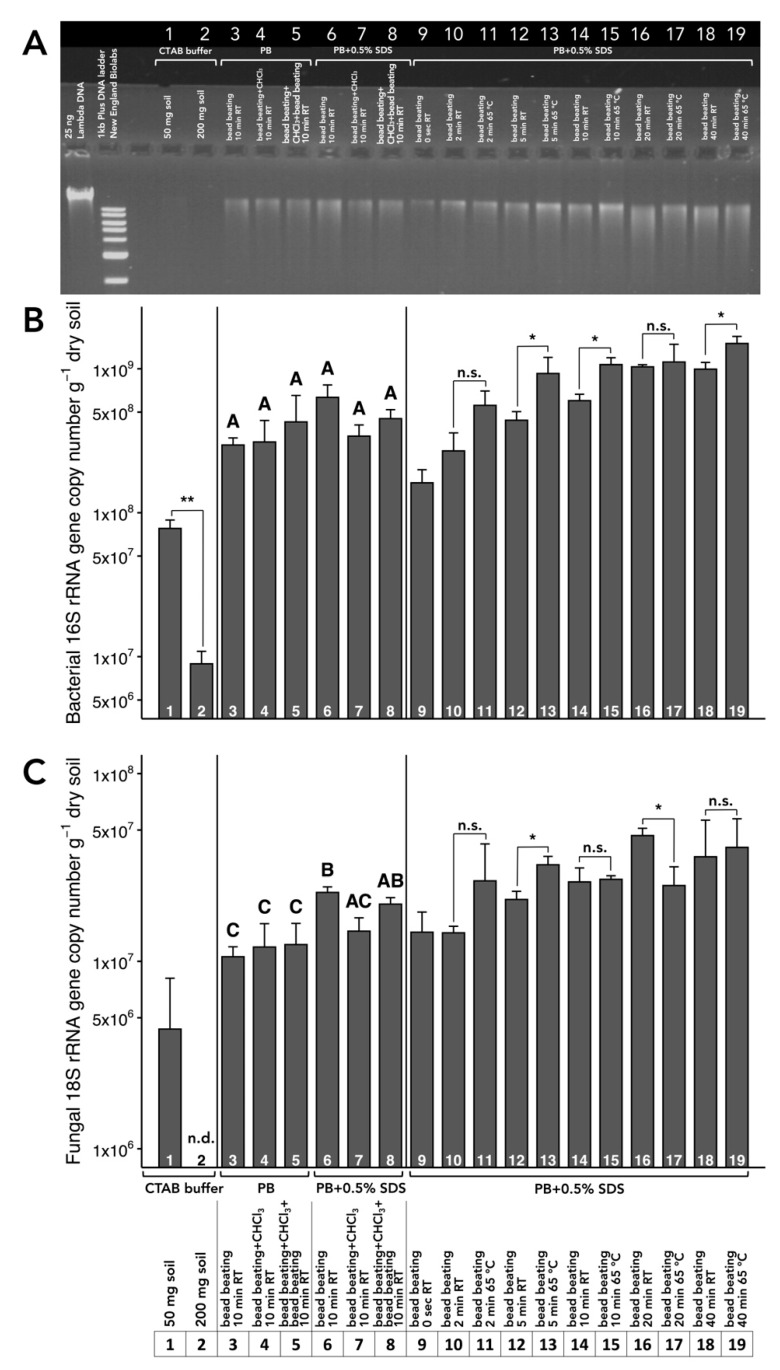
Comparison of DNA obtained with different extraction protocols and copy number of rRNA genes determined by qPCR. (**A**) Separation of extracted DNA in 1.7% agarose gel. Samples loaded onto the gel were pooled from three technical replicates shown in Figure S1. (**B**) Abundance of bacterial 16S rRNA. (**C**) Abundance of fungal 18S rRNA. Differences between two groups of samples (e.g., 1 and 2 or 10 and 11) were tested using student’s t-test (* *p* < 0.05). Differences among more than two groups of samples (e.g., 3 to 8) were tested using ANOVA with Tukey HSD. Different uppercase letters indicate statistically significant differences at *p* < 0.05. n.d. = not detectable; n.s. = no statistically significant differences; PB = phosphate buffer; RT = room temperature; SDS = sodium dodecyl sulfate.

**Figure 4:**
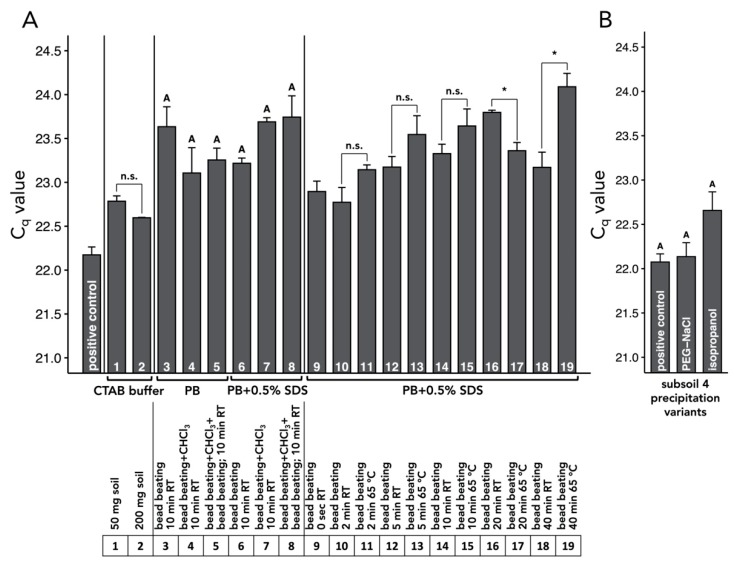
DNA amplification inhibition test of DNA extracts from subsoil using different extraction methods (see 2.4. DNA amplification inhibition test for methodological details) (**A**) and subsoil 4 using polyethylene glycol (PEG)-NaCl and isopropanol precipitation (**B**). Differences between two groups of samples (e.g., 1 and 2 or 10 and 11) were tested using student’s t-test (* *p* < 0.05). Differences among more than two groups of samples (e.g., 3 to 8) were tested using ANOVA with Tukey HSD. Different uppercase letters indicate statistically significant differences at *p* < 0.05. C_q_ = quantification cycle; n.s. = no statistically significant differences; CTAB = cetyltrimethylammonium bromide; PB = phosphate buffer; RT = room temperature; SDS = sodium dodecyl sulfate.

Extraction from 50 and 200 mg subsoil using a cetyltrimethylammonium bromide (CTAB)-based protocol with polyethylene glycol (PEG)-NaCl precipitation resulted in low total DNA yield (Figure 3 A). Likewise, the yield of DNA of soil bacteria and fungi was comparatively low (Figure 3B,C). We observed that the extraction of 200 mg soil resulted in lower recovery of bacteria (*p* = 0.0072) than 50 mg soil (Figure 3B), and fungal DNA was not detectable when DNA was extracted from 200 mg soil (Figure 3C). Compared to the CTAB-based protocol, the usage of 1 M PB increased total DNA yield, as well as the recovery of soil bacteria and fungi (Figure 3). The comparison of three different cell lysis methods (i) bead beating, (ii) bead beating + chloroform, and (iii) bead beating + chloroform + bead beating (Figure 1), prior to DNA extraction using PB with/without 0.5% sodium dodecyl sulfate (SDS) revealed that bead beating prior to extraction in PB with 0.5% SDS resulted in the largest total DNA yield (Figure 1). The cell lysis method (iii) (bead beating + chloroform + bead beating), followed by washing with PB with 0.5% SDS, resulted in similar total DNA yield as bead beating (Figure 3), but consumed more time and resources and did not reduce the effect of PCR inhibitors on amplification (Figure 4A).

### 3.2. Optimization of Incubation Temperature and Time

The optimization of the incubation temperature and time was performed using bead beating, with a subsequent washing using PB with 0.5% SDS. Incubating the soil/PB suspension at 65 °C generally increased total DNA yield as compared to room temperature (RT) (Figure 3). At the same time, however, incubation at 65 °C increased the effect of co-extracted PCR inhibitors (Figure 4A). The optimal incubation time at which the greatest DNA yield with comparatively low degradation was achieved was 10 min for both incubation temperatures, whereas bacterial and fungal recovery was greater at 65 °C than at RT (*p* = 0.010) (Figure 3). Therefore, we selected bead beating of 200 mg freeze-dried subsoil, followed by a 10-min washing using PB with 0.5% SDS, performed at 65 °C (Supplementary File S1) as the optimal extraction protocol.

### 3.3. DNA Extraction from Different Subsoils

**Figure 5:**
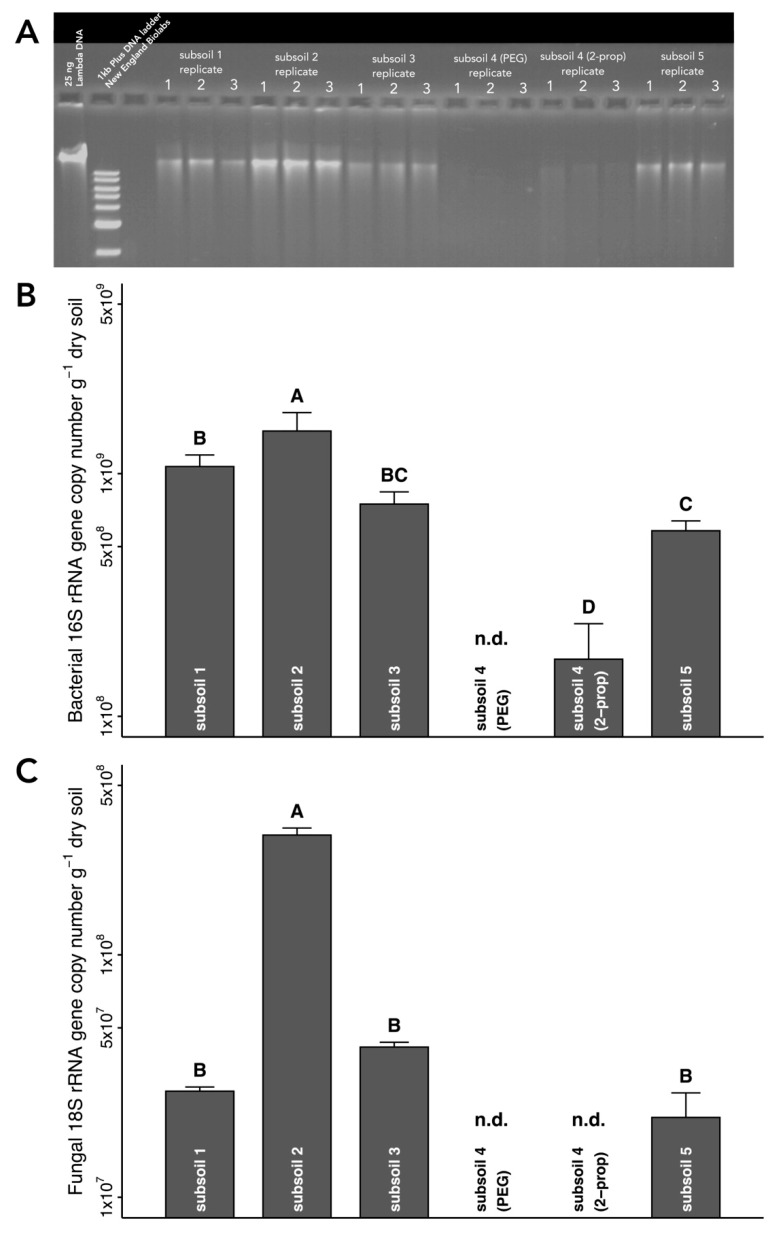
Extraction of DNA from different subsoils using the optimized protocol. (**A**) Separation of DNA in 1.7% (w/v) agarose gel. (**B**) Abundance of bacterial 16S rRNA. (**C**) Abundance of fungal 18S rRNA. DNA extracted from subsoils 1, 2, 3 and 5 was precipitated with PEG, while DNA extracted from subsoil 4 was precipitated with both PEG and isopropanol. Differences among groups were tested using ANOVA with Tukey HSD. Different uppercase letters indicate statistically significant differences at *p* < 0.05. n.d. = not detectable. PEG = polyethylene glycol (PEG)-NaCl precipitation; 2-prop = isopropanol precipitation.

Our optimized extraction protocol for DNA from subsoil (Supplementary File S1) was tested using five different subsoils of different depths and soil characteristics (Table S1). We successfully extracted DNA from subsoils 1, 2, 3, and 5 and were able to quantify soil bacteria and fungi in these extracts using qPCR (Figure 5). Furthermore, visual assessment of extracted DNA in agarose gels as well as qPCR data indicated that our optimized protocol is suitable for these types of subsoil (Figure 5). For subsoil 4, we obtained no supernatant after centrifugation of soil suspension in PB. Even increasing the volume of PB to 500 µL has not allowed us to extract sufficient quantities of DNA for the quantification of bacteria and fungi from subsoil 4 (Figure 5). The replacement of isopropanol as a precipitation agent by PEG-NaCl increased the DNA yield (Figure 5A), but simultaneously appeared to enhance PCR inhibition, although this effect was not supported statistically (Figure 4B). Isopropanol precipitation of DNA from subsoil 4 enabled us to quantify soil bacteria (Figure 5B), whereas soil fungi were still not detectable by qPCR (Figure 5C). The reliability of the optimized extraction protocols was confirmed by an interlaboratory comparison between the University of Goettingen, Germany and the University of Florida, USA (Figure S2).

## 4. Discussion

DNA extraction using a CTAB-based protocol yielded a low amount of DNA when 50 mg soil per mL buffer was used and almost no detectable DNA when 200 mg soil was used (Figure 3). Investigations on the effect of pH on the adsorption of DNA to clay minerals have shown that adsorption capacity decreased at higher pH [24,36]. Recently, Hou et al. [47] demonstrated that clay minerals modified with CTAB have a greater capacity for DNA adsorption and intercalation of DNA into the clay galleries. This mechanism may contribute to the overall low performance of CTAB-based protocols on clay soils.

Commercial kits for the extraction of DNA from soil are available, but their performance has often been criticized as unsatisfactory. For instance, the effectiveness of a commercial kit for releasing bound DNA from clay loam soil was called into question by Emmons et al. [48], in a study investigating the persistence of human DNA in grave soil; Vishnivetskaya et al. [29] demonstrated that the DNA content of bacterial taxa in soil DNA extracted using certain commercial kits is biased, and Lim et al. [49] showed that an in-house method outperformed a commercial kit in the extraction of bacterial DNA from sand. To compensate for the shortcomings of commercial kits, using a combination of two kits has been exploited. Dimitrov et al. [50] employed successive extractions of the same sample with two commercially available kits to increase yields of DNA from soils with clay content as high as 36.7%. In a complementary approach, Yamanouchi et al. [51] extracted soil DNA using a commercial kit and processed the extract with another kit to improve the quality of DNA. Antony-Babu et al. [52], however, used two commercial kits for DNA extraction from soil sequentially in this way, yet the quality of DNA was not adequate. Washing soil samples with diluted EDTA five times prior to DNA extraction, and washing DNA bound to the columns of the kit with a concentrated solution of guanidine thiocyanate up to five times was necessary to obtain DNA of adequate quality. For some soils, an additional extraction with a mixture of phenol and chloroform was necessary [52].

Apart from the high costs of commercial kits, especially when two kits are used sequentially, the fundamental drawback of using commercial kits is that the constitution of key components is unknown and their future availability is uncertain. This hampers the replication and continuation of research relying on such kits. For instance, in 2003, Braid et al. [53] developed a method for the removal of PCR inhibitors from soil DNA by flocculation with aluminum ammonium sulfate, based on Ultra-Clean Soil DNA Purification kit (Mo Bio Labs, Solana Beach, CA, USA). In 2005, van den Boogert et al. [54] optimized the use of the kit for the extraction of soil DNA for the diagnosis of *Synchitrium endobioticum*. In 2010, van Gent-Pelzer et al. [55] extended the applicability of the method to fresh wart tissue and optimized its sensitivity. In 2009, Gonzales-Franco et al. [56] developed a method for the extraction of Actinomycetes DNA from soil, based on the same kit with additional heating and bead beating steps. In 2013, all these protocols became obsolete, because the soil DNA extraction kit on which they were based was removed from the market. The protocol developed in this work will not suffer this fate, because it does not use proprietary reagents.

Reports of the effect of PEG versus isopropanol precipitation on DNA yield and co-extraction of contaminants like humic acids are contradictory, as summarized by Arbeli and Fuentes [57]. It was suggested that differences among precipitation protocols likely account for these inconclusive results [57]. For environmental samples with very low microbial biomass, the selection of an appropriate precipitation agent is important for the successful recovery of DNA. Precipitation of DNA with PEG is more selective than precipitation with isopropanol [57]; it was therefore used for the removal of contaminants from DNA extracted from microbial sediments [58] and PCR inhibitors from soil DNA [59]. While some authors reported the efficient removal of PCR inhibitors from DNA by PEG precipitation, others were less successful. For instance, Yeates at al. [60] had to strongly dilute extracted DNA before PCR and Cullen and Hirsch [61] observed brown humic substances co-precipitating with DNA; the suitability of DNA for amplification was equal to DNA precipitated with isopropanol [61]. We assume that the PEG concentration used in these works, namely 10% and 15%, was too high. Arbeli and Fuentes [57] showed in their elegant study that the concentration of humic substances in DNA solution was 5-times higher after precipitation with 10% PEG, as compared to DNA precipitated with 5% PEG. In their work, 5% was the lowest PEG concentration at which no loss of DNA was observed. Five percent was also the lowest PEG concentration traditionally used to precipitate DNA in the presence of 0.5 M NaCl [62]. However, choosing the lowest suitable concentration from a tested series does not lead to a robust protocol, because a slightly lower concentration or the use of PEG with a slightly different length distribution might reduce the yield. Indeed, losses of DNA after precipitation with 5% PEG have been reported [63]. We therefore chose 6.7% PEG for our protocol.

Among five subsoils tested in this work, four subsoils generated sufficient amounts of DNA to be detected in agarose gels, but the extraction of subsoil 4 failed (Figure 5). Replacement of PEG-NaCl precipitation with isopropanol precipitation led to the successful extraction of DNA from subsoil 4 (Figure 5). It should be noted, however, that the substitution of PEG with isopropanol increased the co-extraction of PCR inhibitors as well (Figure 5B). This finding agrees with previous reports that isopropanol precipitation increased the amount of co-extracted humic acids and other contaminants, as compared to precipitation with PEG [57,59]. In cases without a detectable recovery of DNA, carriers such as polyacrylamide [64], carrier DNA [65], or glycogen [66] may be added to soil extracts prior to DNA precipitation.

The inclusion of SDS in the PB increased DNA yield (Figure 3). SDS is a strong anionic detergent that lyses cells [67,68] and is routinely used in soil DNA extraction protocols (e.g., [31]), either as an alternative to mechanical cell disintegration by bead beating [69] or in combination with bead beating [60,70]. While developing a DNA extraction method for marine sediments, Gray and Herwig [71] found that 1 min bead beating, followed by incubation at 70 °C in the presence of lysis buffer containing SDS, resulted in high yields of high molecular weight DNA, but they had to purify the DNA using a commercial kit before amplification. The authors rationalized that the bead beating step was necessary for partial cell lysis and homogenization of the sample while the 70 °C incubation completed the lysis. They also observed that prolonging the bead-beating step caused DNA shearing. In another work [72], mechanical disintegration was found to be the key factor affecting the efficiency of DNA extraction from soil. While SDS has been used to denature proteins (e.g., [39]) and lyse tissues and cells in the laboratory [67,68], chloroform was used in soil microbiology to lyse microbial cells directly in soil [73]. Due to the fact that certain soil bacteria were reported to survive SDS treatment and bead beating [74], we investigated whether treatment with chloroform prior to bead beating improves the DNA recovery from soil. The results showed that chloroform pre-treatment did not improve the yield of DNA when the extraction buffer contained SDS (Figure 3). Therefore, we chose the bead beating of freeze-dried soil, with subsequent washing with PB containing SDS as the extraction method. In our experience, the incubation of freeze-dried soil at 65 °C in PB with 0.5% SDS for 10 min resulted in high DNA yield with minimal degradation. Dry clays and adsorbed DNA may experience greater electrostatic interaction as DNA molecules change configuration and become closely packed under dry conditions, resulting in greater negative charge density [28]. In our study, incubation for 10 min might have provided enough time for rewetting and weakening such electrostatic interactions. Furthermore, the amount of DNA and other compounds released from cells in the presence of SDS have been observed to dramatically increase around ten minutes’ elapsed time [68]. It is likely that the initial homogenization step, along with the bead beating step, mechanically lysed some cells and additionally homogenized the clay, which facilitated penetration of the lysis buffer.

## 5. Conclusions

We assessed different lysis procedures in combination with phosphate buffers for the extraction of DNA from a range of clay-rich subsoils. The best recovery was achieved after homogenization by bead beating, followed by an extraction with 1 M phosphate buffer and 0.5% SDS at 65 °C. The DNA extraction protocol for subsoils is suitable for downstream molecular analysis, and is robust as well as reproducible across laboratories.

## Supplementary Materials

Supplementary materials can be found at https://www.mdpi.com/2076-2607/8/4/532/s1.

## References

[1] Tebrügge F., Düring R.-A. (1999). Reducing tillage intensity—A review of results from a long-term study in Germany. Soil Tillage Res..

[2] Sosa-Hernández M.A., Leifheit E.F., Ingraffia R., Rillig M.C. (2019). Subsoil Arbuscular Mycorrhizal Fungi for Sustainability and Climate-Smart Agriculture: A Solution Right under Our Feet?. Front. Microbiol..

[3] Frelih-Larsen A., Hinzmann M., Ittner S. (2018). The ‘Invisible’ Subsoil: An Exploratory View of Societal Acceptance of Subsoil Management in Germany. Sustainability.

[4] Holden P.A., Fierer N. (2005). Microbial Processes in the Vadose Zone. Vadose Zone J..

[5] von Lützow M., Kögel-Knabner I., Ekschmitt K., Matzner E., Guggenberger G., Marschner B., Flessa H. (2006). Stabilization of organic matter in temperate soils: Mechanisms and their relevance under different soil conditions—A review. Eur. J. Soil Sci..

[6] Fontaine S., Barot S., Barré P., Bdioui N., Mary B., Rumpel C. (2007). Stability of organic carbon in deep soil layers controlled by fresh carbon supply. Nature.

[7] Xiang S.-R., Doyle A., Holden P.A., Schimel J.P. (2008). Drying and rewetting effects on C and N mineralization and microbial activity in surface and subsurface California grassland soils. Soil Biol. Biochem..

[8] Chabbi A., Kögel-Knabner I., Rumpel C. (2009). Stabilised carbon in subsoil horizons is located in spatially distinct parts of the soil profile. Soil Biol. Biochem..

[9] Fierer N., Allen A.S., Schimel J.P., Holden P.A. (2003). Controls on microbial CO2 production: A comparison of surface and subsurface soil horizons. Glob. Chang. Biol..

[10] Oehl F., Sieverding E., Ineichen K., Ris E.A., Boller T., Wiemken A. (2005). Community structure of arbuscular mycorrhizal fungi at different soil depths in extensively and intensively managed agroecosystems. New Phytol..

[11] Sagova-Mareckova M., Zadorova T., Penizek V., Omelka M., Tejnecky V., Pruchova P., Chuman T., Drabek O., Buresova A., Vanek A. (2016). The structure of bacterial communities along two vertical profiles of a deep colluvial soil. Soil Biol. Biochem..

[12] Kautz T., Perkons U., Athmann M., Pude R., Köpke U. (2013). Barley roots are not constrained to large-sized biopores in the subsoil of a deep Haplic Luvisol. Biol. Fertil. Soils.

[13] Hobley E., Bauke S., Steffens M., Amelung W., Kögel-Knabner I. (2018). Hyperspectral Imaging of Soil Cores Reveals Greatest C Storage in Subsoil Biopores.

[14] Uksa M., Schloter M., Endesfelder D., Kublik S., Engel M., Kautz T., Köpke U., Fischer D. (2015). Prokaryotes in Subsoil—Evidence for a Strong Spatial Separation of Different Phyla by Analysing Co-occurrence Networks. Front. Microbiol..

[15] Lavahun M.F.E., Joergensen R.G., Meyer B. (1996). Activity and biomass of soil microorganisms at different depths. Biol. Fertil. Soils.

[16] Taylor J.P., Wilson B., Mills M.S., Burns R.G. (2002). Comparison of microbial numbers and enzymatic activities in surface soils and subsoils using various techniques. Soil Biol. Biochem..

[17] Zhang B., Penton C.R., Xue C., Quensen J.F., Roley S.S., Guo J., Garoutte A., Zheng T., Tiedje J.M. (2017). Soil depth and crop determinants of bacterial communities under ten biofuel cropping systems. Soil Biol. Biochem..

[18] Sosa-Hernández M.A., Roy J., Hempel S., Kautz T., Köpke U., Uksa M., Schloter M., Caruso T., Rillig M.C. (2018). Subsoil arbuscular mycorrhizal fungal communities in arable soil differ from those in topsoil. Soil Biol. Biochem..

[19] Tebbe C.C., Vahjen W. (1993). Interference of humic acids and DNA extracted directly from soil in detection and transformation of recombinant DNA from bacteria and a yeast. Appl. Environ. Microbiol..

[20] Ranjard L., Poly F., Combrisson J., Richaume A., Nazaret S. (1998). A single procedure to recover DNA from the surface or inside aggregates and in various size fractions of soil suitable for PCR-based assays of bacterial communities. Eur. J. Soil Biol..

[21] Frostegård Å., Courtois S., Ramisse V., Clerc S., Bernillon D., Le Gall F., Jeannin P., Nesme X., Simonet P. (1999). Quantification of Bias Related to the Extraction of DNA Directly from Soils. Appl. Environ. Microbiol..

[22] Hurt R.A., Robeson M.S., Shakya M., Moberly J.G., Vishnivetskaya T.A., Gu B., Elias D.A. (2014). Improved yield of high molecular weight DNA coincides with increased microbial diversity access from iron oxide cemented sub-surface clay environments. PLoS ONE.

[23] Goring C.A.I., Bartholomew W.V. (1952). Adsorption of Mononucleotides, Nucleic Acids, and Nucleoproteins by Clays. Soil Sci..

[24] Greaves M.P., Wilson M.J. (1969). The adsorption of nucleic acids by montmorillonite. Soil Biol. Biochem..

[25] Ogram A., Sayler G.S., Gustin D., Lewis R.J. (1988). DNA adsorption to soils and sediments. Environ. Sci. Technol..

[26] Khanna M., Stotzky G. (1992). Transformation of Bacillus subtilis by DNA bound on montmorillonite and effect of DNase on the transforming ability of bound DNA. Appl. Environ. Microbiol..

[27] Ogram A.V., Mathot M.L., Harsh J.B., Boyle J., Pettigrew C.A. (1994). Effects of DNA Polymer Length on Its Adsorption to Soils. Appl. Environ. Microbiol..

[28] Pietramellara G., Dal Canto L., Vettori C., Gallori E., Nannipieri P. (1997). Effects of air-drying and wetting cycles on the transforming ability of DNA bound on clay minerals. Soil Biol. Biochem..

[29] Vishnivetskaya T.A., Layton A.C., Lau M.C.Y., Chauhan A., Cheng K.R., Meyers A.J., Murphy J.R., Rogers A.W., Saarunya G.S., Williams D.E. (2014). Commercial DNA extraction kits impact observed microbial community composition in permafrost samples. FEMS Microbiol. Ecol..

[30] Waring B.G., Álvarez-Cansino L., Barry K.E., Becklund K.K., Dale S., Gei M.G., Keller A.B., Lopez O.R., Markesteijn L., Mangan S. (2015). Pervasive and strong effects of plants on soil chemistry: A meta-analysis of individual plant ‘Zinke’ effects. Proc. R. Soc. B Biol. Sci..

[31] Zhou J., Bruns M.A., Tiedje J.M. (1996). DNA recovery from soils of diverse composition. Appl. Environ. Microbiol..

[32] Hurt R.A., Qiu X., Wu L., Roh Y., Palumbo A.V., Tiedje J.M., Zhou J. (2001). Simultaneous Recovery of RNA and DNA from Soils and Sediments. Appl. Environ. Microbiol..

[33] Sagova-Mareckova M., Cermak L., Novotna J., Plhackova K., Forstova J., Kopecky J. (2008). Innovative methods for soil DNA purification tested in soils with widely differing characteristics. Appl. Environ. Microbiol..

[34] He J., Xu Z., Hughes J. (2005). Pre-lysis washing improves DNA extraction from a forest soil. Soil Biol. Biochem..

[35] Ogram A., Sayler G.S., Barkay T. (1987). The extraction and purification of microbial DNA from sediments. J. Microbiol. Methods.

[36] Cai P., Huang Q., Zhang X., Chen H. (2006). Adsorption of DNA on clay minerals and various colloidal particles from an Alfisol. Soil Biol. Biochem..

[37] Brandfass C., Karlovsky P. (2008). Upscaled CTAB-Based DNA Extraction and Real-Time PCR Assays for Fusarium culmorum and F. graminearum DNA in Plant Material with Reduced Sampling Error. Int. J. Mol. Sci..

[38] Chourey K., Jansson J., VerBerkmoes N., Shah M., Chavarria K.L., Tom L.M., Brodie E.L., Hettich R.L. (2010). Direct cellular lysis/protein extraction protocol for soil metaproteomics. J. Proteome Res..

[39] Chatterjee A., Moulik S.P., Majhi P.R., Sanyal S.K. (2002). Studies on surfactant–biopolymer interaction. I. Microcalorimetric investigation on the interaction of cetyltrimethylammonium bromide (CTAB) and sodium dodecylsulfate (SDS) with gelatin (Gn), lysozyme (Lz) and deoxyribonucleic acid (DNA). Biophys. Chem..

[40] Beule L., Corre M.D., Schmidt M., Göbel L., Veldkamp E., Karlovsky P. (2019). Conversion of monoculture cropland and open grassland to agroforestry alters the abundance of soil bacteria, fungi and soil-N-cycling genes. PLoS ONE.

[41] Beule L., Lehtsaar E., Corre M.D., Schmidt M., Veldkamp E., Karlovsky P. (2020). Poplar Rows in Temperate Agroforestry Croplands Promote Bacteria, Fungi, and Denitrification Genes in Soils. Front. Microbiol..

[42] Lane D.J. (1991). 16S/23S rRNA Sequencing. Nucleic Acid Techniques in Bacterial Systematics.

[43] Muyzer G., de Waal E.C., Uitterlinden A.G. (1993). Profiling of complex microbial populations by denaturing gradient gel electrophoresis analysis of polymerase chain reaction-amplified genes coding for 16S rRNA. Appl. Environ. Microbiol..

[44] Vainio E.J., Hantula J. (2000). Direct analysis of wood-inhabiting fungi using denaturing gradient gel electrophoresis of amplified ribosomal DNA. Mycol. Res..

[45] Eynck C., Koopmann B., Grunewaldt-Stoecker G., Karlovsky P., von Tiedemann A. (2007). Differential interactions of Verticillium longisporum and V. dahliae with Brassica napus detected with molecular and histological techniques. Eur. J. Plant Pathol..

[46] R Core Team (2017). R: A Language and Environment for Statistical Computing.

[47] Hou Y., Wu P., Huang Z., Ruan B., Liu P., Zhu N. (2014). Successful intercalation of DNA into CTAB-modified clay minerals for gene protection. J. Mater. Sci..

[48] Emmons A.L., DeBruyn J.M., Mundorff A.Z., Cobaugh K.L., Cabana G.S. (2017). The persistence of human DNA in soil following surface decomposition. Sci. Justice.

[49] Lim H.J., Choi J.-H., Son A. (2017). Necessity of purification during bacterial DNA extraction with environmental soils. Environ. Health Toxicol..

[50] Dimitrov M.R., Veraart A.J., de Hollander M., Smidt H., van Veen J.A., Kuramae E.E. (2017). Successive DNA extractions improve characterization of soil microbial communities. PeerJ.

[51] Yamanouchi K., Takeuchi M., Arima H., Tsujiguchi T. (2019). Development of a method to extract protozoan DNA from black soil. Parasite Epidemiol. Control.

[52] Antony-Babu S., Murat C., Deveau A., Tacon F.L., Frey-Klett P., Uroz S. (2013). An improved method compatible with metagenomic analyses to extract genomic DNA from soils in Tuber melanosporum orchards. J. Appl. Microbiol..

[53] Braid M.D., Daniels L.M., Kitts C.L. (2003). Removal of PCR inhibitors from soil DNA by chemical flocculation. J. Microbiol. Methods.

[54] Van den Boogert P.H.J.F., van Gent-Pelzer M.P.E., Bonants P.J.M., De Boer S.H., Wander J.G.N., Lévesque C.A., van Leeuwen G.C.M., Baayen R.P. (2005). Development of PCR-based Detection Methods for the Quarantine Phytopathogen Synchytrium endobioticum, Causal Agent of Potato Wart Disease. Eur. J. Plant Pathol..

[55] Van Gent-Pelzer M.P.E., Krijger M., Bonants P.J.M. (2010). Improved real-time PCR assay for detection of the quarantine potato pathogen, Synchytrium endobioticum, in zonal centrifuge extracts from soil and in plants. Eur. J. Plant Pathol..

[56] Gonzalez-Franco A.C., Robles-Hernandez L., Nuñez-Barrios A., Strap J.L., Crawford D.L. (2009). Molecular and cultural analysis of seasonal actinomycetes in soils from Artemisia tridentata habitat. Phyton (Buenos Aires).

[57] Arbeli Z., Fuentes C.L. (2007). Improved purification and PCR amplification of DNA from environmental samples. FEMS Microbiol. Lett..

[58] Purdy K.J., Embley T.M., Takii S., Nedwell D.B. (1996). Rapid Extraction of DNA and rRNA from Sediments by a Novel Hydroxyapatite Spin-Column Method. Appl. Environ. Microbiol..

[59] LaMontagne M.G., Michel F.C., Holden P.A., Reddy C.A. (2002). Evaluation of extraction and purification methods for obtaining PCR-amplifiable DNA from compost for microbial community analysis. J. Microbiol. Methods.

[60] Yeates C., Gillings M.R., Davison A.D., Altavilla N., Veal D.A. (1998). Methods for microbial DNA extraction from soil for PCR amplification. Biol. Proced. Online.

[61] Cullen D.W., Hirsch P.R. (1998). Simple and rapid method fordirect extraction of microbial DNA fromsoil for PCR. Soil Biol. Biochem..

[62] Dunn I.S., Blattner F.R. (1987). Charons 36 to 40: Multi enzyme, high capacity, recombination deficient replacement vectors with polylinkers and polystuffers. Nucleic Acids Res..

[63] Paithankar K.R., Prasad K.S. (1991). Precipitation of DNA by polyethylene glycol and ethanol. Nucleic Acids Res..

[64] Gaillard C., Strauss F. (1990). Ethanol precipitation of DNA with linear polyacrylamide as carrier. Nucleic Acids Res..

[65] Graham F.L., van der Eb A.J. (1973). A new technique for the assay of infectivity of human adenovirus 5 DNA. Virology.

[66] Green M.R., Sambrook J. (2016). Precipitation of DNA with Ethanol. Cold Spring Harb. Protoc..

[67] Woldringh C.L. (1970). Lysis of the cell membrane of Escherichia coli K12 by ionic detergents. Biochim. Biophys. Acta (BBA)—Nucleic Acids Protein Synth..

[68] Woldringh C.L., Van Iterson W. (1972). Effects of Treatment with Sodium Dodecyl Sulfate on the Ultrastructure of Escherichia coli. J. Bacteriol..

[69] Kabir S., Rajendran N., Amemiya T., Itoh K. (2003). Real-time quantitative PCR assay on bacterial DNA: In a model soil system and environmental samples. J. Gen. Appl. Microbiol..

[70] Miller D.N., Bryant J.E., Madsen E.L., Ghiorse W.C. (1999). Evaluation and Optimization of DNA Extraction and Purification Procedures for Soil and Sediment Samples. Appl. Environ. Microbiol..

[71] Gray J.P., Herwig R.P. (1996). Phylogenetic analysis of the bacterial communities in marine sediments. Appl. Environ. Microbiol..

[72] Plassart P., Terrat S., Thomson B., Griffiths R., Dequiedt S., Lelievre M., Regnier T., Nowak V., Bailey M., Lemanceau P. (2012). Evaluation of the ISO Standard 11063 DNA Extraction Procedure for Assessing Soil Microbial Abundance and Community Structure. PLoS ONE.

[73] Brookes P.C., Landman A., Pruden G., Jenkinson D.S. (1985). Chloroform fumigation and the release of soil nitrogen: A rapid direct extraction method to measure microbial biomass nitrogen in soil. Soil Biol. Biochem..

[74] Moré M.I., Herrick J.B., Silva M.C., Ghiorse W.C., Madsen E.L. (1994). Quantitative cell lysis of indigenous microorganisms and rapid extraction of microbial DNA from sediment. Appl. Environ. Microbiol..

