# Supplementary material for: Improved Protocol for DNA Extraction from Subsoils Using Phosphate Lysis Buffer
Source: Microorganisms. 2020 Apr 7;8(4):532. doi: 10.3390/microorganisms8040532 (PMC7232467; doi:10.3390/microorganisms8040532)
Supplement: Supplementary file 1 [file microorganisms-08-00532-s001.zip › microorganisms-767267-supplementary 3/2020_Guerra_Beule_Supplementary_tables_figures.docx]

**Table S1.** Sampling details and soil biochemical properties of the five different subsoils.

|  | **sample details** | | | | | |
| --- | --- | --- | --- | --- | --- | --- |
| **sample ID** | **sampling Location** | **sampling depth (cm)** | **pH^1^** | **organic C (g kg-2)^2^** | **total N (g kg-2)^2^** | **C:N ratio** |
| Subsoil 1 | Dornburg, Germany | 60 | 7.35 | 0.41 | 0.07 | 5.86 |
| Subsoil 2 | Nordhausen, Germany | 60 | 7.02 | 0.42 | 0.06 | 7.00 |
| Subsoil 3 | Goettingen, Germany | 70 | 8.12 | 0.14 | 0.03 | 4.67 |
| Subsoil 4 | Wendhausen, Germany | 120 | 7.73 | 3.02 | 0.18 | 16.52 |
| Subsoil 5 | Goettingen, Germany | 150 | 7.60 | 0.16 | 0.03 | 5.33 |

^1^ determined in 1:4 H2O; ^2^ determined using a CN analyzer (Elementar Vario El, Elementar Analysis Systems GmbH, Hanau, Germany).

| 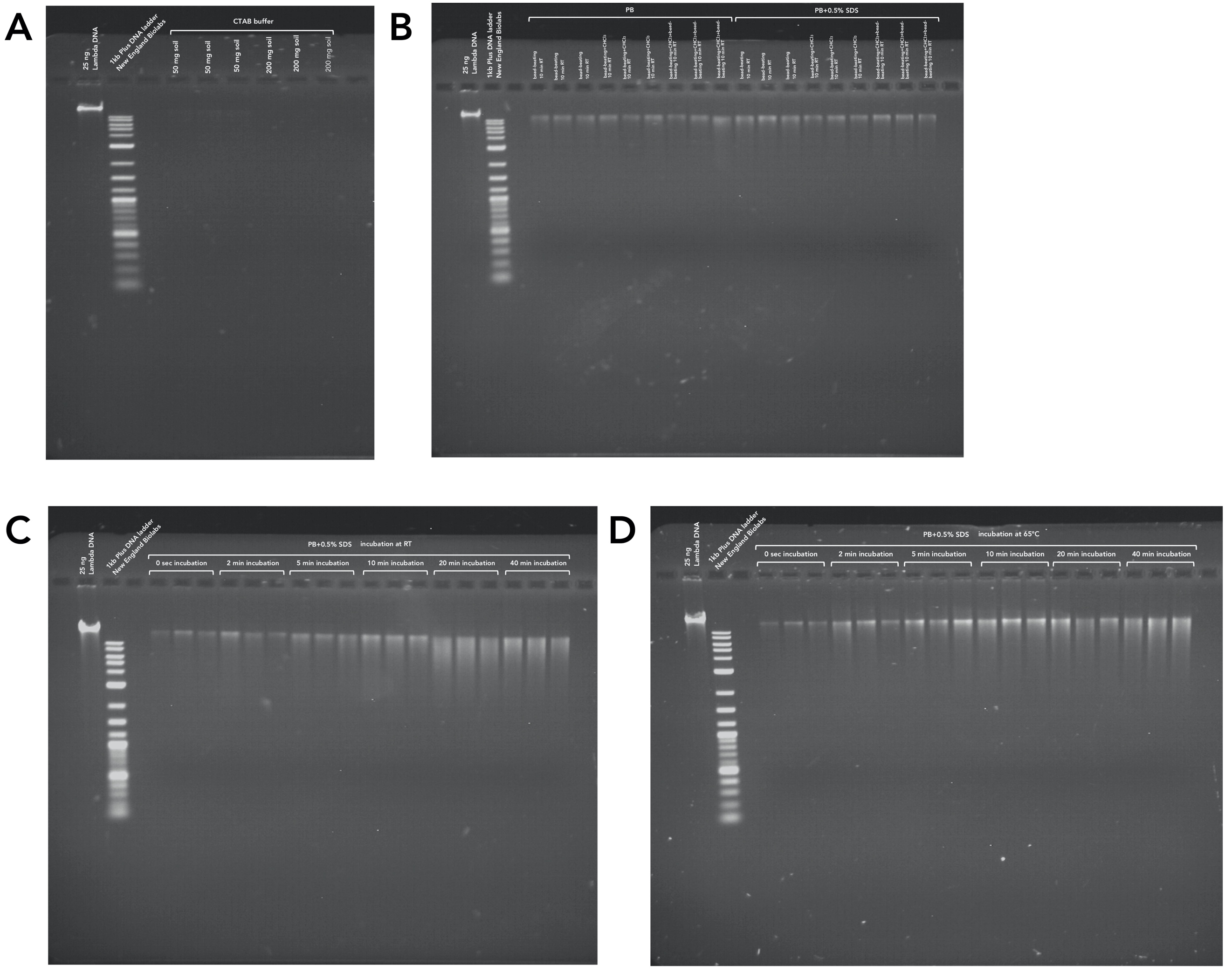 |
| --- |

**Figure S1.** 1.7% (w/v) agarose gels with 3 µL DNA extract showing the three technical replicates of the CTAB-based extraction protocol (**A**), the comparison of the lysis method using 1M PB with/without 0.5% SDS (**B**), the incubation time gradient at RT (**C**) and 65°C (**D**). CTAB = cetyltrimethylammonium bromide; PB = phosphate buffer; RT = room temperature; SDS = sodium dodecyl sulfate.

| 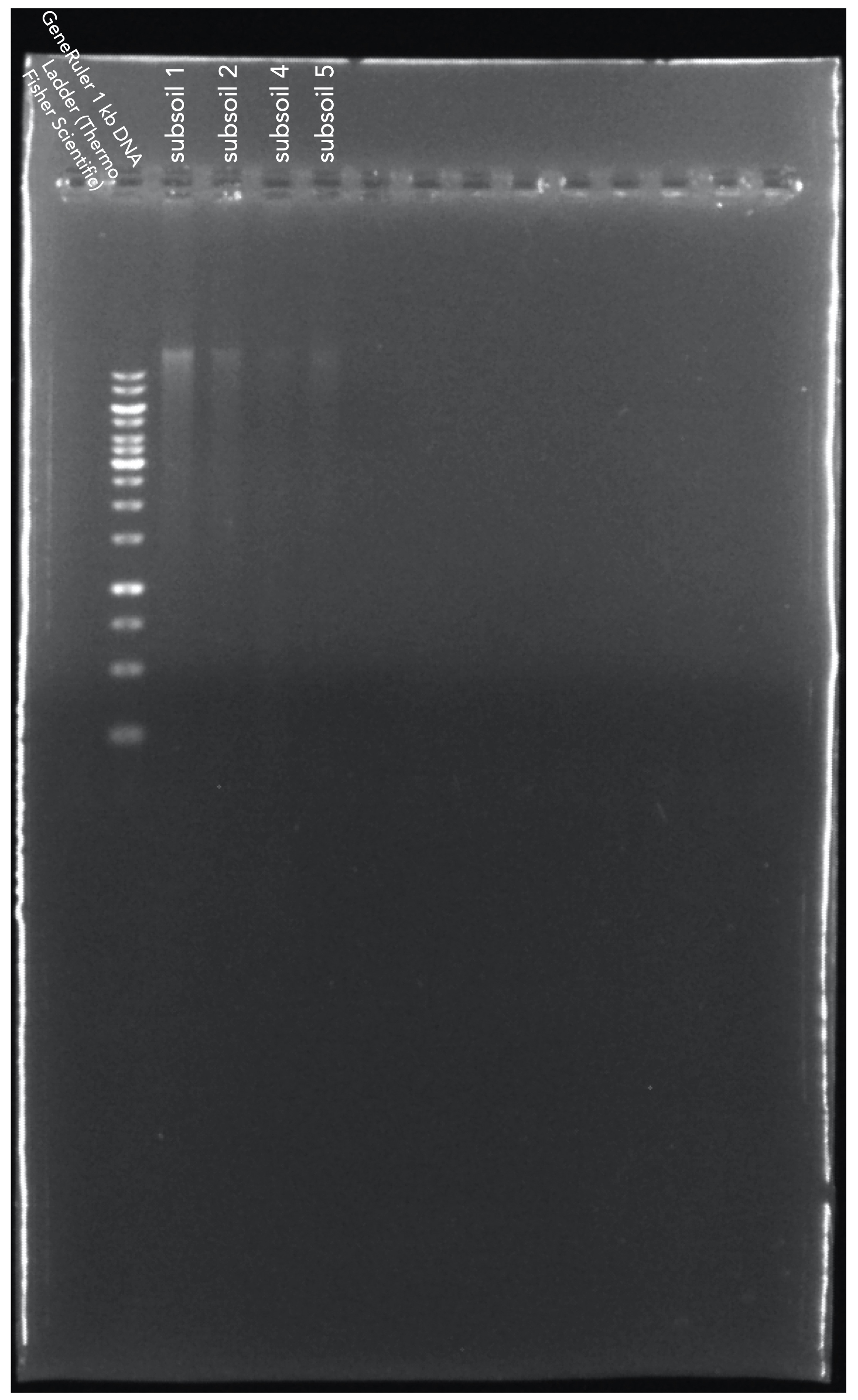 |
| --- |

**Figure S2.** 1% (w/v) agarose gel with 10 µL DNA extract from five different subsoils using the optimized extraction method. Subsoil 3 was not extracted due to limited sample volume. DNA extracted from subsoil 4 were precipitated using isopropanol.
