# Supplementary material for: Improved Protocol for DNA Extraction from Subsoils Using Phosphate Lysis Buffer
Source: Microorganisms. 2020 Apr 7;8(4):532. doi: 10.3390/microorganisms8040532 (PMC7232467; doi:10.3390/microorganisms8040532)
Supplement: Supplementary file 1 [file microorganisms-08-00532-s001.zip › microorganisms-767267-supplementary 3/Supplementary_file_1.docx]

**Protocol for DNA extraction from clay-rich subsoils**

Weigh in 200 ± 5 mg freeze-dried soil into a 2-mL tube.

1. Homogenize the sample by bead beating using 3 tungsten carbide beads (ø 3 mm) for 1 min at 25 Hz using a swing mill (e.g. MM 400, Retsch, Germany).
2. Add 250 µL PB buffer^1^ with 0.5% SDS (w/v) and vortex for 10 sec at max speed.
3. Incubate the suspension at 65°C for 10 min and shake the samples every 60 sec for 5 sec.
4. Centrifuge for 1 min at 10,000 rpm and transfer 90 µL of the supernatant into a new 2-mL tube (supernatant will contain bubbles).
5. Add 810 µL ddH_2_O (1:10 dilution) and subsequently 900 µL phenol^2^ and shake the samples briefly.
6. Centrifuge for 10 min at 10,000 rpm and transfer 800 µL of the upper phase into a new 2-mL tube.
7. Add 800 µL chloroform:isoamylalcohol (24:1 (v/v)), incubate 10 min on ice and centrifuge for 10 min at 10,000 rpm.
8. Transfer 700 µL of the upper phase into a new 1.5 mL-tube.
9. Add 700 µL chloroform:isoamylalcohol (24:1), incubate 10 min on ice and centrifuge for 10 min at 10,000 rpm.
10. Transfer 600 µL of the upper phase into a new 1.5 mL-tube containing 200 µL 30% PEG 6000 and 100 µL 5M NaCl.
11. Shake the mixture and incubate at room temperature for 20 min.
12. Centrifuge for 15 min at max. speed.
13. Wash the pellet with 500 µL 80% EtOH (v/v) twice and centrifuge for 5 min at max speed in-between each washing before discarding the supernatant. Supernatant should be removed by pipetting.
14. Dry the pellet using vacuum centrifugation at 30°C for 20 minutes or until it is dry under a sterile bench.
15. Add 50 µL TE buffer, vortex or flick the samples to release the pellet from the tube wall and incubate at 42°C for 2h (e.g. in a water bath).

**Notes addressing the solutions:**

^1^ PB buffer: 1 M Na_2_HPO_4_ and 1 M NaH_2_PO_4_, blended to achieve pH 7.2.

^2^ Phenol: redistilled, in TE buffer equilibrated, pH 7.5 to 8.0
